# Nanotechnology Tolls the Bell for Plastic Surgeons

**Published:** 2013-06

**Authors:** Zeinab Salehahmadi, Fatemeh Hajiliasgari

**Affiliations:** 1 Department of Information Technology, Bushehr University of Medical Sciences, Bushehr, Iran;; 2 Deputy of Health, Tehran University of Medical Sciences, Tehran, Iran; Director of Information Technology and Department, Academy of Russian Sciences, Tajikestan

**Keywords:** Nanotechnology, Nanosurgery, Plastic surgery

## Abstract

Nanotechnology is an emerging discipline, having power to revolutionarize every scientific field to a very deep level which previously thought to be a science fiction. Having a great potential to beneficially change the way a disease is diagnosed, treated and prevented, nanotechnology practically impacts on state of the art healthcare technologies and plays a crucial role in changing the field of surgery. Surgeons are constantly looking for minimally invasive ways to treat their patients, as recovery is faster when a lesser trauma is inflicted upon a patient, scarring is lessened and there are usually fewer complications in the aftermath of the operation. Through nanotechnology, tiny biosensors could be constructed which could take these factors into account, thus shortening the patient recovery period and saving hospitals money, reducing infection rates within the hospital, reducing the waiting lists for operation and allowing doctors to treat more patients in the same period of time. This review employs a thematic analysis of online series of academic papers focuses on the potentials of nanotechnology in surgery, especially in plastic surgery and addresses the possible future prospects of nanotechnology in this field.

## INTRODUCTION

**Fig. 1:**
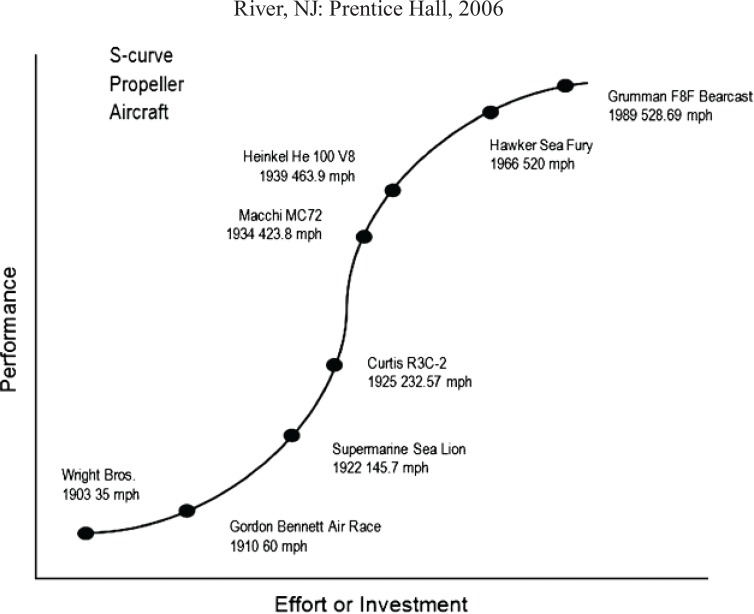
Speed of propeller aircraft on y-axis as a function of effort/investment. This demonstrates the S curve concept where there is ultimately a leveling off of performance despite continued effort. Figure adapted from Foster LE, Nanotechnology: Science, Innovation, & Opportunity, Chapter 1, p.7 Upper Saddle.^11^

The prefix “nano” derives from the Greek word for “dwarf”. One is 80,000 nm wide, and a red blood cell is approximately 7000 nm wide. Atoms are smaller than 1 nm, whereas many molecules including some proteins range between 1 nm and larger.^1^ The conceptual underpinnings of nanotechnologies were first laid out in 1959 by the physicist Richard Feynman in his lecture, there’s plenty of room at the bottom. Feynman explored the possibility of manipulating materials at the scale of individual atoms and molecules, imagining the whole of the Encyclopedia Britannica written on the head of a pin and foreseeing the increasing ability to examine and control matter at the nano scale. The term nanotechnology was not used until 1974, when Norio Taniguchi, a researcher at the University of Tokyo, used it to refer to the ability to engineer materials precisely at the nanometer level. The primary driving force for miniaturization at that time came from the electronics industry, which aimed to develop tools to create smaller (and therefore faster and more complex) electronic devices on silicon chips. Furthermore, at IBM in the United States, a technique called electron beamlithography was used to create nanostructures and devices as small as 40 to 70 nmin the early 1970s. “Nanotechnology” refers to the design, production and application of structures, devices or systems at the incredibly small scale of atoms and molecules–the “nanoscale**”.**^2^ Nanotechnology is the manipulation or self-assembly of individual atoms, molecules, or molecular clusters into structures to create materials and devices with new or vastly different properties. Nanotechnology can work from the top down (which means reducing the size of the smallest structures to the nanoscale e.g. photonics applications in nanoelectronics and nanoengineering) or the bottom up (which involves manipulating individual atoms and molecules into nanostructures and more closely resembles chemistry biology).^3^ Recently the use of nanoprticles (NPs) has come an attractive platform in biomedical applications and have had a great impact on the medical community. Trends towards refining surgical methods to produce minimal trauma to the body in line with enhanced recovery programs have already begun. Smaller incisions, laparoscopic surgery, microsurgery operating under the microscope, robotics and implants have already produced a move towards the miniaturization of surgical techniques.^4^ Applications of nanobiotechnology are already beginning to show great impact on the practice of conventional medicine in practice today. The National Cancer Institute has recognized the great importance of nanotechnology in the future of all types of benign and malignant cancers and has delivered large grants for achievements in this field for the future. The Office for Science and Technology in the UK has recommended more research into the toxicological aspects of nanotechnology which will undoubtedly pose challenges to researchers as we delve yet deeper into the realms of this field.^5^ Despite this, it appears certain that advances in nanomedicine offer the possibility of new and exciting opportunities in NPs detection, diagnosis and treatment of surgical disease. This new technology was born out of necessity and out of the physical limits come across by computer chip manufacturers who have been laboring for decades to make computer chips smaller and faster. As the logic gates have shrunk to the atomic scale, Newtonian physics has taken a backseat to quantum mechanical effects. At quantum scales, completely new properties emerge, and the need to characterize these new properties resulted in the explosion of research in nanotechnology.^6^ Jurvetson pointed out, ‘‘If we took your entire genetic code, the entire biologic program that resulted in your cells, organ, body, and mind and burned it into a CD, it would be smaller than Microsoft Office, resulting in a 750-MB file.’’ Even more humbling, if genetic redundancies were removed and the file was compressed, it would only be 60 MB.^7^ As memory storage devices become smaller, technological discontinuity and the S curve in Lynn Foster’s excellent book on nanotechnology, Gerald Gallwas used the Wright brothers 1903 invention of the propeller airplane as an illustration of the S curve concept.^8^ He showed graphically how the original top speed of the aircraft was a about 35 mph (Figure 1). As time (and effort/investment) into this new technology progresses on the x-axis, its performance on the y-axis (top speed in this case) closely follows the shape of an S curve, ultimately approaching a physical limit. When this limit is reached, a discontinuity is created, and a new innovation is needed to significantly increase performance. The invention of the jet engine bridged that discontinuity, improved performance, and also started its own S curve. This example is mentioned because the lesson of the S curve is relevant to medicine and plastic surgery. There are a tremendous number of devices, materials, and procedures that are used every day by plastic surgeons, which have or will soon reach the peak of their development cycles on the S curve. Suture is one example. It has been used for hundreds of years. Recent innovations have involved advances in the material, adding barbs to the suture, or impregnating it with antibiotics. These improvements have led to more controlled absorption (or decreased biodegradetion), faster skin closure, and fewer potential infections. But as in the case of propeller planes, there is clearly a limit. The performance of suture has scarcely changed in several hundred years nor has what we have asked it to do. Another example is the use of unaided visual inspection and cutting instruments in surgery. As Singhal *et al.* point out: ‘‘The surgeon used cutting instruments, his or her eyes and hands, intuition, and experience, no intraoperative tools or devices have successfully improved the surgeon’s ability to find and remove a tumor in over a half a century”.^9^ Although somewhat hyperbolic, Singhal’s point is well taken. What is being proposed is that in addition to sutures and cutting instruments, there are dozens of everyday instruments, and materials that are being used by plastic surgeons that have reached the limit of their performance on the S curve.^10^

The purpose of this article is to deal with the topic of nanotechnology and to discuss its relevance to surgery in general and plastic surgery in particular. Nanotechnology will be defined, and some important historical milestones discussed. Common applications of nanotechnology in various medical and surgical subspecialties will be reviewed. Future applications of nanotechnology to plastic surgery will be examined. 

*Nano-Surgery*

The potential for the use of nanotechnology in surgery is huge. Surgeons are constantly looking for minimally invasive ways to treat their patients, as recovery is faster when a lesser trauma is inﬂicted upon a patient, scarring is lessened and there are usually fewer complications in the aftermath of the operation. Surgeons operate on the macro scale, and have made great advances over the last century. Surgery has evolved from ‘‘no more science than butchery’’ to a highly respected science, and even an art. Through nanotechnology, tiny biosensors could be constructed which could take these factors into account, thus shortening a patients recovery period and saving hospitals expenditure, reducing nosocomial infection rates, reducing the waiting lists for operation and allowing doctors to treat more patients in the same period of time. A surgical nanobot, programmed by a human surgeon could act as an autonomous on-site surgeon inside the human body. Various functions such as searching for pathology, diagnosis and removal or correction of the lesion by nanomanipulation can be performed and coordinated by an on-board computer. The performance of surgical blades can be enhanced signiﬁcantly when micro-structured hard metal is coated with diamond and processed. Major advantages of the diamond nano-layers in this application are low physical adhesion to materials or tissues and chemical/biological inertness. In addition, diamond has a low friction coefﬁcient decreasing the penetration force necessary. Nano-needles prepared from silicon and attached to an atomic force microscope can be used to penetrate the nucleus of living cells to deliver molecules and may be even used to carry out cell surgery. Nano-wires can be engineered to sense and pick up molecular markers of cancer cells and pinpoint the changes in the genetics of cancer. The nanometer-sized cantilevers are extremely sensitive and can detect single molecules of DNA or protein. Thus providing fast and sensitive detection methods for cancer related molecules. Nano-particle contrast agents are being developed for tumor detection purposes. Super paramagnetic nano-particles are used for magnetic resonance imaging. One of the greatest achievements of nanotechnology in surgery will be what we call the “ideal graft” that is, biocompatible and durable “repairs” of parts of the body like arteries, joints or even organs. At first, these repairs will be used for healing, but soon afterwards, they will be used for transcendence: to enhance current human abilities.^12^ Non-composite nano-materials and nano-technological approaches cover a wide range of surgical topics, including self-assembling nano-fibres for haemostasis,^13^ conduits for nerve repair,^14^ nanofabricated drains^15^ and means of studying and affecting in vivo processes such as neuro-protection and neuro-modulation in real time.^16^ ‘Smart’ instruments have sensors embedded in them, which provide the surgeons with data on internal conditions, as they are performing procedures. These are sensors that have embedded technology within the medical device instrument, which allows the surgeon to get an internal perspective of the surgery or procedure they are performing. Smart sensors are currently used by surgeons when performing complicated procedures, but more complex applications for such devices are presently being developed for the market.^17^

*Nano-Robots*

Robots have recently been introduced to undertake basic surgical procedures, and with the help of nano-biotechnology, another dimension of robotics has been developed. This is commonly known as nano-robots or nano-bots. These miniature robots are so small that they can be introduced into the body either through the vascular system or through catheters, and with external guidance and monitoring by the surgeon they can perform precise intracellular surgery, which would not be possible with the human hand. The exterior of a nano-robot will likely be constructed of carbon atoms in a diamondoid structure because of its inert properties and strength. Super-smooth surfaces will lessen the likelihood of triggering the body’s immune system, allowing the nano-robots to go about their business unimpeded. Glucose or natural body sugars and oxygen might be a source for propulsion and the nano-robot will have other biochemical or molecular parts depending on its task.^18^ The preliminary goal is to use various biological elements-whose function at the cellular level creates a motion, force or a signal-as nano-robotic components that perform the same function in response to the same biological stimuli but in an artificial setting. Nano-robots would constitute any passive or active structure (nano-scale) capable of actuation, sensing, signalling, information processing, intelligence and swarm behavior at nano-scale. These functionalities could be illustrated individually or in combinations by a nanorobot.^19^ Some of the characteristic abilities that are desirable for a nano-robot to function are: Swarm intelligence-decentralization and distributive intelligence, cooperative behavior, an architecture enabling instant access to nano-robots and their control and maintenance.^20^ A surgical nanobot, programmed by a human surgeon, could act as an autonomous on-site surgeon inside the human body. Various functions such as searching for pathology, diagnosis and removal or correction of the lesion by nano-manipulation can be performed and coordinated by an on-board computer while maintaining contact with the supervising surgeon via coded ultrasound signals. Nano-robots will have the capability to perform precise and refined intracellular surgery, which is beyond the capability of manipulations by the human hand. Nano-robots will be scavengers for atherosclerotic plaque. Minimally invasive micro-robots will be used instead of stents inside arteries, for repairs that are currently being performed laparoscopically. Nano-particle-assisted surgery already illuminates cancers, so that surgeons can completely remove them, or even visually scan the body for metastasis. Nano-particle-based local drug delivery will also soon help diagnose and treat cancer in a more cell-to- cell fashion, that is, detect and treat cancer in the very first stage of the disease. Individualized therapies, with cancer-specific nano-particle vehicles, will be available and enhanced by personalized genomics for every patient. Investigations are being performed on creating artificial flagella designed to mimic natural bacteria in both size and swimming technique and on nanobots for retinal surgery. One goal nanobot is to overturn the basic paradigm of today’s medicine, and to shift from a treatment model to a prevention model through the use of in-body sensors, which check for and kill pathogens before the patient has any symptoms.^21^ A rapidly vibrating (100 Hz) micropipette with a <1 μm tip diameter has been used to completely cut dendrites from single neurons without damaging cell viability. Axotomy of roundworm neurons was performed by femto-second laser surgery, after which the axons functionally regenerated.^22^

*Catheters for Minimally Invasive Surgery*

Catheters are small tubes which are inserted into the body cavity to inject or drain fluids or to keep a passageway clear. One of the issues associated with catheters is thrombus formation on the surface of these devices. Nano-materials, e.g. carbon nano-tubes, have been successfully added to catheters used in minimally invasive surgery to increase their strength and flexibility and reduce their thrombogenic effect. Improved electrostatic properties and dense surface topology caused by the nucleation function of the carbon nano-tubes have probably contributed to the antithrombotic property. Catheters can also be coated with silver NPs to give them antibacterial properties and prevent surface biofilm formation.^23^

*Nano-Plastic Surgery*

Nanotechnology is being hailed as the “next industrial revolution”. Nano-materials are now found in hundreds of products, from cosmetics to clothing to food products. In plastic surgery, it is anticipated that this new technology may be instrumental. Nanotechnology is a novel technology which will likely lead to advancements in the art and science of plastic surgery. Special nano-enhanced materials can potentially be used to promote healing, control infection, restore function, and aid nerve regeneration and rehabilitation. Micro-electromechanical system nanotechnology offers the ability to develop miniaturized implants for use in the treatment of numerous conditions. Electrical stimulation has several potential applications in plastic surgery, ranging from the restoration of facial animation following tumor ablation or trauma to serving an adjunctive role in myoelectric prostheses. A micro-electromechanical systems–based electrochemical stimulation method has been developed that is potentially implantable, clinically efficacious, and durable which might be used to treat a multitude of clinical conditions and provide a platform for the management of nervous dysfunction syndromes.^24^

*Breast Implants and Capsular Contracture*

The interaction of implanted materials with the surrounding tissue can be detrimental to the implant and/or the host. This interaction is often the result of an over-exuberant immune response secondary to infection, hematoma, or foreign body reaction. Capsular contracture is a common example of this in plastic surgery. Barr *et al.* have found that fibroblasts are affected by micro- and nano-topographies influencing the long-term biointegration of devices such as breast implants. They cite a study that demonstrated that cell filopodia are capable of sensing ‘‘nanoislands down to a size of 10 nm.’’ The implication is that the nanoscale architecture could have an impact on the body’s immune response to a breast implant and the subsequent development of capsular contracture. Barr *et al.* in their study examined the surface architecture of the shells of breast implant materials approved for use in the United Kingdom. They found remarkable variability in the surface characteristics. Specifically, they found that smooth implants have a ‘‘shallow, regular, 5-Km period rippled texture’’ that is directional and thought to be due to how the implants are manufactured. This surface architecture is thought to be responsible for the higher rate of capsular contracture of these smooth implants by stimulating fibroblasts to form capsules. In contrast, Biocell and Siltex surfaces with 100 to 200 Km deep, random surface features are thought to anchor implants in position and reduce contracture. Barr *et al.* conclude wisely that implant surfaces require modification to enhance their biocompatibility. Many questions are raised by their study. Could custom nano-topographies or coatings with integrated and controlled release antibiotics into implant shells be the solution to capsular contracture? Could anti-inflammatory agents be introduced onto the surface of implants to inhibit the development of contracture? What is the ideal surface architecture required to minimize the inflammatory response and biofilm formation? The answers to these questions may hold the key to future innovations in implant surfaces.^25^

*Tissue Engineering*

Tissue engineering has been defined as ‘the application of principles and methods of engineering and life sciences towards fundamental understanding of structure function relationships in normal and pathological mammalian tissues and the development of biological substitutes to restore, maintain or improve tissue function’.^26^ Tissue-engineered products typically are a combination of three components, i.e. isolated cells, an extracellular matrix and signal molecules, such as growth factors. Nanotechnology provides new possibilities for the extracellular matrix, often referred to as the scaffold. The extracellular matrix serves three primary roles. First, it facilitates the localization and delivery of cells in the body. Second, it defines and maintains a three-dimensional space for the formation of new tissues with an appropriate structure. Third, it guides the development of new tissues with appropriate function. The interaction of the cells and extracellular matrix is of great importance for the intended function of the final product. Thus, several of the principles described above for implantable materials in orthopedics and dentistry, providing greater biocompatibility and/or stimulating the in growth of cells into the material in situ, are at least equally valid for tissue engineering scaffolds. Micro and nano-structured surfaces of scaffold materials have important beneficial effects on cell adhesion and proliferation. Control of (nano) porosity is essential to obtain three-dimensional constructs with the appropriate desired properties. Also, the possibility to tailor mechanical characteristics, matching the target tissue as closely as possible, leads to increased possibilities for successfully functioning tissue-engineered products. In a recent study on “Nasal reconstruction using tissue engineered constructs” done (2013), the gold standard for reconstruction after rhinectomy or severe trauma to the nose has been deployed and it includes transposition of autologous mucosal flaps plus autologous cartilage grating and coverage using a skin flap. The advancement of such approach is dependent on the dissemination of scientific information into the clinical community, regarding the engineering of tissues such as mucosa, skin, and cartilage. ^27^

*Wound Dressing*

Metallic silver is known for its anti-infective properties, which are effective against a wide range of bacteria and microorganisms. A nano-porous silver powder, which can be applied to a range of products, has been developed. Smaller particles give a greater surface area, and, therefore, a better anti-infective surface. Also, less silver is required overall, so there is less risk of any toxic side effects. Applications for nano-silver coatings on medical devices include implants, indwelling catheters and wound dressings, and for burns and other chronic wounds. The nano-silver enters the wound through body fluids and can reportedly kill bacteria in 30 min. Each dressing can last for several days depending on the thickness of the layer of nano-silver.^28^ The effect of organoclay quantity on the structural, swelling, physical and mechanical properties of nano-composite hydrogel wound dressing was investigated. The results showed that the nanocomposite hydrogels could meet the essential requirements for the reasonable wound dressing with some desirable characteristics such as relatively good swelling, appreciated vapor transmission rate, excellent barrier against microbe entrance and mechanical properties. The results also indicated that the quantity of the clay added to the nano-composite hydrogel is the key factor in obtaining such suitable properties required for wound dressing.^29^

*Reconstruction*

Stubinger *et al.* used nano-structured HA-based biomaterial to perform sinus floor elevations on 20 patients and found that the material had excellent tissue biocompatibility and noted that within 6 months, new trabecular bones were formed without any need for autogenous bone. The histologic analysis showed that the nano-enhanced biomaterial acted ‘‘as a strong osteoconductive bone substitute with no evidence of an ongoing marked foreign body reaction.’’^30^ Adamopoulos and Papadopoulos in their recent article discussed the manufacturing techniques and properties of biomaterials and bio-ceramics based on nanotechnology. Bioceramics are used widely in dentistry and among maxillofacial surgeons to replace lost teeth, fill jaw defects, mandibular reconstruction, and temporomandibular joint surgery.^31^ It was noted that nano-phase HA has been demonstrated to have improved osteointegrative properties, and 3D porous nano-HA scaffolds with bone marrow seeds showed cell adherence, proliferation, and differentiation lending promise to reconstructing bony defects. By more closely simulating the nanostructure of natural bones, the prospect for greater osteointegration, more natural mechanical properties, and less immune response and greater control of cell responses nano-phase HA can make better implants. This has significant implications for craniofacial surgery. Nano-machine that functions to create proteins countless times within every human cell, it has no central nervous system. It has only its naturally embedded programming encoded in its 3D-folded conformation and selected by nature over millennia. With technological advances in computer science and engineering, this evolutionary design could be replicated over a timescale orders of magnitude smaller to perform specific functions such as target cancer cells, remove arterial plaques, making a wound environment more favorable to healing by removing toxins and bacterial by products, construct soft tissues or organs. Aptamers are examples of nucleic acids whose 3D conformation is selected for functionality in a high throughput manner, which compresses millions of years of evolution into hours. Aptamers are being used as receptors for small molecules, as biosensors, and as therapeutic agents. Nano-robots and aptamers could be used to help regenerate or reconstruct individual axons and nerves through specialized conduits eliminating the need for nerve grafts. They could perform multiple, seamless, submillimeter arterial, venous, and lymphatic anastomosis; be used to repair cleft palates and repair cleft lips in utero or to prevent craniosynostosis entirely; be embedded in dressings to perform wound debridement and rapidly help close large open wounds; or be injected into tendon sheaths and break up tendon adhesions after tendon repair.^32^

*Future of Nano-Plastic Surgery*

Applications of nano-biotechnology are already beginning to show great impact on the practice of conventional medicine in practice today. All the current modalities that are being used in other fields for imaging, treatment of cancer, and drug delivery can certainly be applied to plastic surgery. Research should and will continue in these areas, however, the unique aspects of nanotechnology raise the question of how these properties are best used to help our patients? Suture or injectable nano-beads could easily be impregnated with steroids, which could then be placed into keloids to release steroid over weeks and months to treat keloids rather than perform repeated painful injections. These nano-beads could also be targeted to keloids or skin cancers using photodynamic therapy where the conjugated NPs is used to selectively target the cells of interest and as the photo sensitizer or drug-delivery agent. Titanium plates for facial fractures could be replaced with biodegradable plates that are coated with fine layers of antibiotics, anti-inflammatory drugs, anesthetic agents, as well as cytokines to stimulate bone healing. This could be done easily and would replace a passive immobilizing device with a more active device that could control pain, decrease infection risk, and help reduce the length patients needed to remain in inter-maxillary fixation in the case of mandible fractures. Breast implants could be modified to not only decrease the rate of capsular contracture by altering their nano-architecture. They could also be coated with antibacterial agents to reduce the risk of infection. They could be made to sense rupture and release a dye that changes the color of a patient’s urine so the patient becomes aware of the rupture. This property might allow patients forgo the current expensive Food and Drug Administration recommendations to obtain an MRI every several years. With the greater strength of many nano-materials, the possibility of rupture could be eliminated entirely. Breast implants could also become more active in breast cancer surveillance and be embedded with proteins that could detect breast cancer cells, treat them, and potentially notify the patient’s oncologist of a local recurrence. Other implantable devices could likewise be packed with useful, active, therapeutic, and screening functions. It is well known that fetal tissue heals without scar. Artificial extracellular matrices have already been synthesized. They can self assemble from simple precursors, and yet display the complex properties of fully functional biologic extracellular matrices. Stem cells grow more favorably in these 3-dimensional (3D) complex matrices than on flat petri dishes. It could be possible to create an artificial fetal in healing wounds and prevent scarring entirely. This environment could be made by packing suture with small nano-machines that deploy upon contact with human tissue and begin the repair of damaged structures to result in perfectly healed wounds. Special nano-enhanced materials are used that could be applied to pressure ulcers to prevent shear, promote healing, and control infection. Quantum dots could be embedded in wound dressings or bandages to detect and speciate an early infection emitting different frequencies for vancomycin-resistant enterococci, *Escherichia coli*, MRSA, *Staphylococcus aureus*, etc. The presence of a clinical infection would prompt the dressing to treat the causative organism with the correct antibiotic using an embedded targeted nano-particle delivery system. This could help eliminate the need for empirical therapy and broad-spectrum antibiotics potentially reducing resistance and systemic side effects. The flow of microvascular anastomosis could be measured in situ, and the information relayed to an external system to alert nursing staff of a pending clot. Taking things one step further, an arterial or venous coupler could be impregnated with antithrombotic agents that could be released when resistance to flow increased above a threshold and signaled a pending clot. Small nano-machines were deployed to break up and destroy clots in fractions sufficiently small to preclude embolization. There is no reason why any of this could not be done in principle. It could be possible to print out soft tissue one day in a manner similar to how 3D cad systems make parts for automobiles. It may be possible to design and build tissues using lithographic techniques in a top down design or to construct tissues using 3D cellular matrices in a bottom up manner. These matrices could then be doped with stem cells to create various skin appendages or tissues types, including cartilage, skin, bone, muscle, and nerve, which could be immunologically neutral and used for ‘‘off-the-shelf’’ reconstruction. Any significant milestones in tissue engineering or nerve regeneration will require multiple disciplines working together in close collaboration. Scientists, biologists, surgeons, physicists, computer scientists, regulatory agencies will no doubt to involve the material. The University of Basel has had an interdisciplinary program in nanotechnology for undergraduates through graduate school for the past decade. The world’s first MD/PhD program in medicine and nanotechnology opens its doors to its first class this year. Other programs will certainly follow to make these seemingly impossible applications commonplace in the years to come. This increases the burden of health and safety on research institutions, medical societies, IRB committees, and individual practitioners. Dermatologists recently called for a wide-ranging panel to advocate for patient safety. We would be wise to heed this call together, researchers, business leaders, scientists, regulators, and consumers must align efforts to address emerging health and safety issues unique to engineered nano-materials to maximize the positive impact of nanotechnology while minimizing and mitigating any and all associated risks.^33^ When any new technology is adopted it can be touted as a panacea. Weldon *et al.* rightly suggest that surgeons should adopt a ‘‘healthy nano-skepticism’’ to counteract much of the ‘‘pseudo-nanoscience’’ and ‘‘nano-fetishism’’ in the literature and popular media. Their criticism that adding the prefix ‘‘nano’’ to many terms can suggest an unwarranted gravity and importance is certainly valid and should be seriously taken.^34^

## CONCLUSION

Advances in nano-biotechnology are revolutionizing our capability to understand biological intricacies and resolve biological and medical problems by developing subtle biomimetic techniques. Preliminary investigations support the potential of nano-biomaterials in orthopedic applications; however, significant advances are necessary to achieve clinical use. The research areas of implanted interfaces, tissue engineering and therapeutics have focused over many years. Nanotechnology has enhanced capability in these various areas. Nanotechnology will help the surgeons’ life easier by use of surgical tools such as nano-needles, nano-surgical blades, better diagnostic imaging and better postoperative period by using precisely targeted delivery system for the medicine. This will help in reducing complications by being able to plan treatment with more precision and leading to better execution of the treatment plan. Advances in plastic surgery will certainly be impacted by this new technology and the full creative weight of the plastic surgery community should be gathered to influence and shape its applications and uses in our field. Safety considerations relating to this new technology should remain paramount and close collaboration with other specialties both within medicine and within the science and engineering community should be enthusiastically sought.

## CONFLICT OF INTEREST

The authors declare no conflict of interest.

## Footenote

Please cite this paper as:

Salehahmadi Z, Hajiliasgari F. Nanotechnology Tolls the Bell for Plastic Surgeons. World J Plast Surg 2013;2(2): 71-80.
